# Supplementary material for: Organic electrochemical neurons and synapses with ion mediated spiking
Source: Nat Commun. 2022 Feb 22;13:901. doi: 10.1038/s41467-022-28483-6 (PMC8863887; doi:10.1038/s41467-022-28483-6)
Supplement: Supplementary file 1 — Supplementary Information [file 41467_2022_28483_MOESM1_ESM.pdf]

Supplementary Information

Organic Electrochemical Neurons and Synapses with Ion Mediated Spiking

Padinhare Cholakkal Harikesh[1,†], Chi-Yuan Yang[1,†], Deyu Tu[1], Jennifer Y. Gerasimov[1], Abdul Manan Dar[1], Adam Armada-Moreira[1], Matteo Massetti[1], Renee Kroon[1], David Bliman[2], Roger Olsson[2,3], Eleni Stavrinidou[1,4], Magnus Berggren[1,4,5], Simone Fabiano[1,4,5]*

[†] Contributed equally.

[1]Laboratory of Organic Electronics, Department of Science and Technology, Linköping University, SE-601 74 Norrköping, Sweden.

[2]Department of Chemistry and Molecular Biology, University of Gothenburg, Gothenburg, SE-412 96 Sweden

[3]Chemical Biology and Therapeutics, Department of Experimental Medical Science, Lund University, Lund, SE-221 84 Sweden

[4]Wallenberg Wood Science Center, Linköping University, SE-601 74 Norrköping, Sweden.

[5]n-Ink AB, Teknikringen 7, 583 30 Linköping, Sweden.

Correspondence should be addressed to: simone.fabiano@liu.se

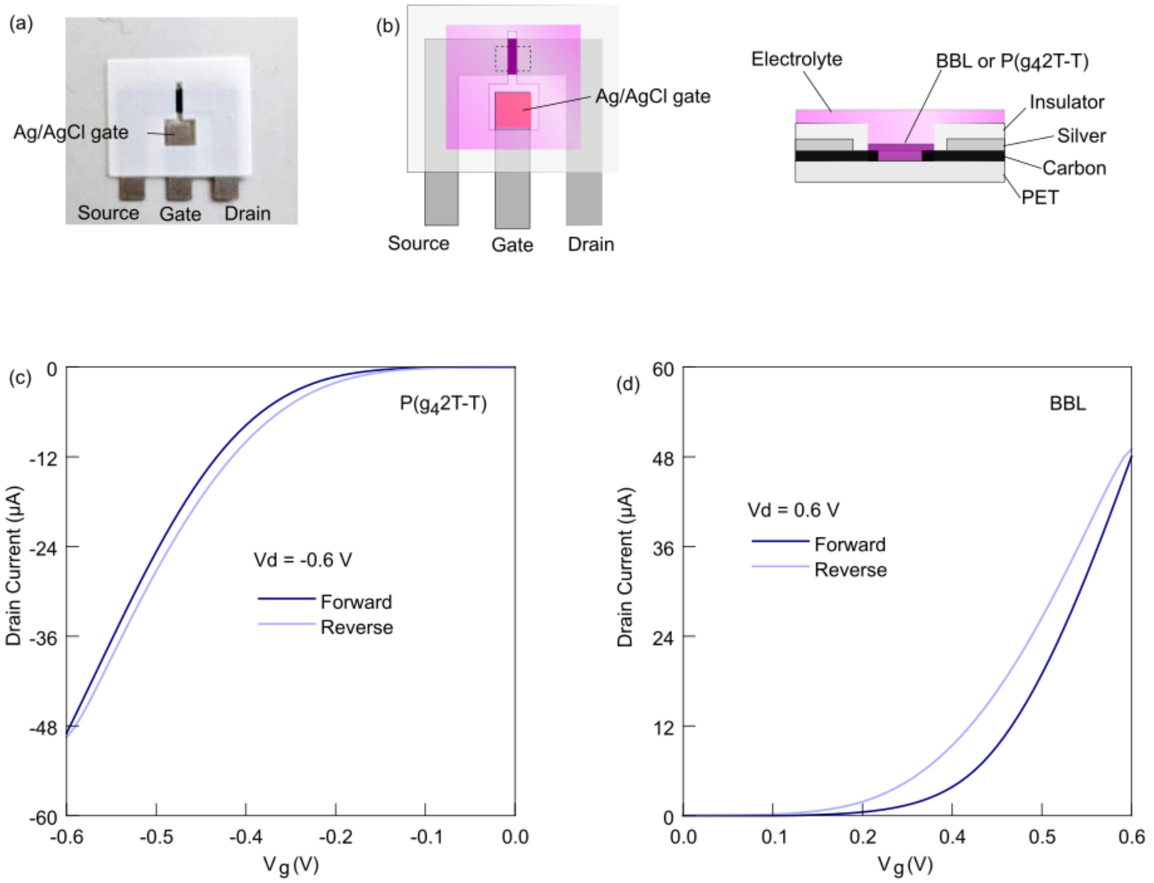

Supplementary Figure 1. (a) Photograph of a single OECT and its (b) lateral device geometry. Transfer characteristics of (c) P($g_4$2T-T) and (d) BBL used for the circuit.

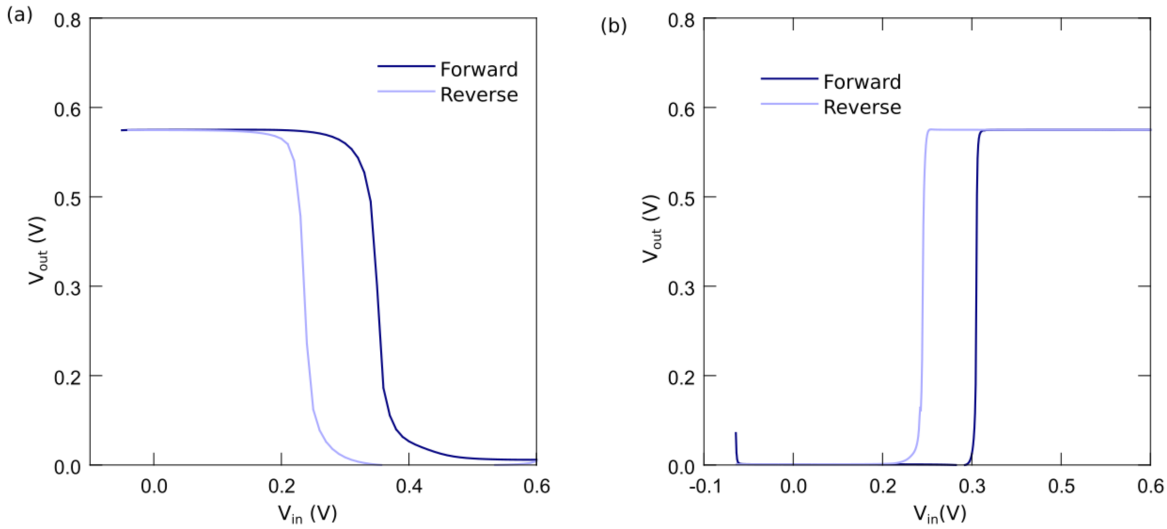

Supplementary Figure 2. Characteristics of the (a) single and (b) two stage inverters.

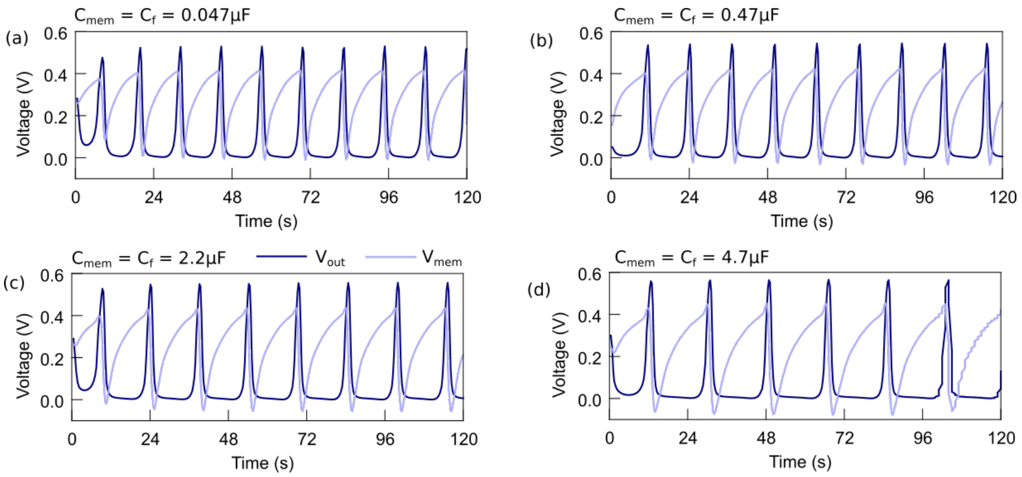

Supplementary Figure 3. Neuron characteristics at various capacitance values and a constant input current of 1 μA

Supplementary Note 1: Improving the firing frequency of OECNs.

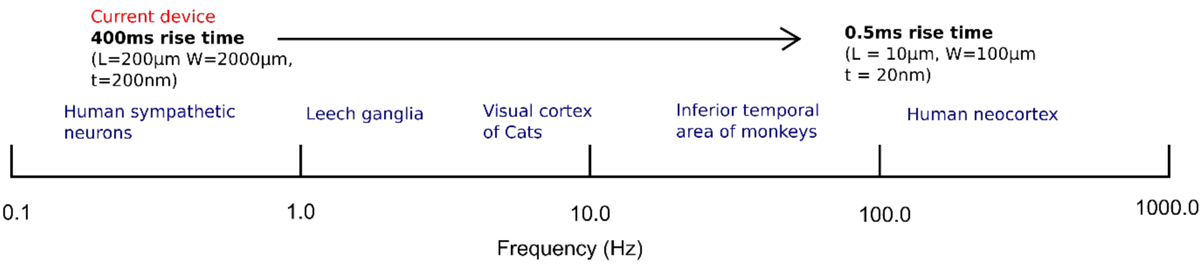

Supplementary Figure 4. Wide frequency range of biological neurons and the device sizes required to achieve similar frequencies in OECNs.

Biological neurons operate in a wide range, with frequencies ranging from < 1 Hz to around 500 Hz. For example, the firing frequency of neurons in the leech ganglia is about 1-5 Hz[1]. The neurons in the primary visual cortex of cats and the inferior temporal area of macaque monkeys fire at around 4 Hz and 20 Hz, respectively[2]. Even human neurons exhibit a wide range of firing frequency. For example, the vasoconstrictor neurons supplying muscle or skin and sudomotor neurons supplying sweat glands have a low firing rate of around 0.5 Hz[3], and the fastest spiking neurons in the human neocortex have a frequency of operation around 500 Hz[4].

The frequency of the OECNs is limited by the response time of the individual transistors and the C_{mem} and C_f capacitors used in the circuit. As the typical rise time of the p- and n-type OECTs used here are 280 ms and 390 ms, the total delay for the 2-stage amplifier with 4 OECTs is ~1.4 s (700 ms delay per stage). In addition to that, there will be a delay introduced by the

resetting OECT (390 ms) and the charging and discharging of C_{mem} and C_f. Hence, even if C_{mem} and C_f were to be omitted from the circuit, the total delay would still be about 2 s corresponding to a maximum theoretical frequency of around 500 mHz. We obtained a maximum frequency of around 300 mHz which is close to this limit. This frequency falls in the lower limit of biological neuron spiking rates (Supplementary Figure 4). To reach the fastest spiking human neurons > 100 Hz, the total delay in the neuron has to be reduced to around 2-3 ms. For example, if the rise time of p-/n-type OECTs is around 0.5 ms, the simulated firing frequency reaches 95 Hz with a 2 nF membrane capacitor and 1 μA input current (Supplementary Figure 9).

The device sizes, capacitances and response times of typical printed n-type OECTs are shown in Supplementary Table 1. Here we take the slower n-type BBL OECT as an example. The total capacitance of the system was calculated using transient current measurements under gate voltage pulses of 0.6 V and -0.6 V with drain and source grounded. The channel capacitance and parasitic capacitance were then estimated using the channel dimensions. We observed that the measured device response time varies linearly with the parasitic capacitance (see Supplementary Figure 5). Extrapolating this, we infer that a 1000× reduction in capacitances (compared to the one used in this study), and hence the total material volume, will be required to obtain response times close to 0.5-1 ms. This would mean a 10× reduction in L (and overlap), W, and thickness of the OECT channel material. This estimation is consistent with our recently reported lithographically-made BBL OECTs with around 1 ms rise times using W = 100 μm, L = 10 μm, and thickness of 20 nm[5].

Supplementary Table 1. Channel dimensions, capacitances and rise times of various printed n-type BBL OECTs. The thickness of the layer is kept at 100-200 nm to obtain similar drain currents of around 50-100 μA at a drain and gate voltage of 0.6 V for all the OECTs

	L (μm)	W (μm)	Overlap (μm)	Total measured Capacitance (μF)	Estimated channel Capacitance (μF)	Estimated parasitic Capacitance (μF)	Measured rise time (ms)
1	63	2000	180	71.2	10.6	60.6	712
**2 (used for the OECNs)**	**200**	**2000**	**100**	**80**	**40**	**40**	**390**
3	56	2000	76	38.8	10.5	28.3	207
4	30	1000	40	35	9.5	25.5	150

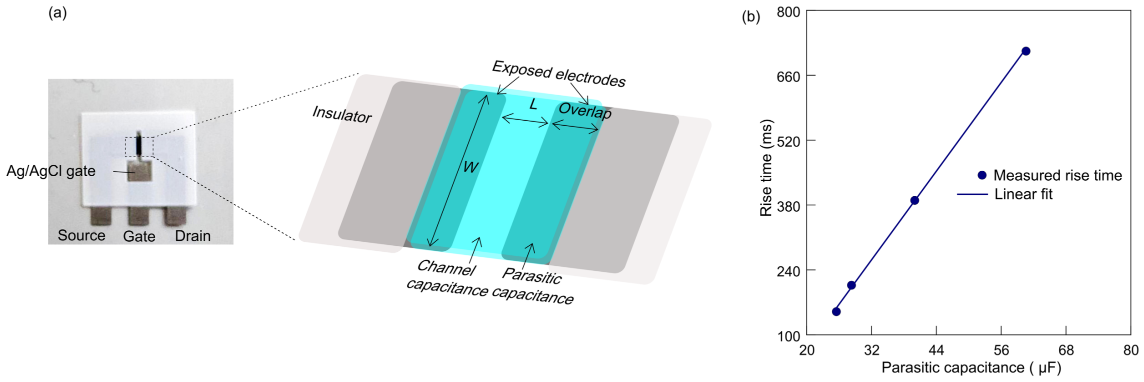

Supplementary Figure 5. (a) Geometry of the OECT. (b) Linear relation between the rise time and parasitic capacitance.

Although such dimensions are not achievable by the screen printing processes adopted in this study, other advanced printing techniques like reverse offset printing can achieve resolution in the range of 1 μm[6], potentially enabling less than 1 ms response times and frequencies higher than 100 Hz. With nanoimprint lithography[7], the channel length can even be shortened to < 100 nm. In addition, the rise times of the transistors can be further reduced by engineering the OECT channel material. For example, the response times of BBL-based OECTs can be reduced down to ~1/10 from 200 ms to 15 ms by mixing BBL with multi-walled carbon nanotubes[8]. The same approach could also be extended to the p-type OECTs. This would then allow even channels with larger dimensions to achieve high frequencies.

Supplementary Note 2: SPICE simulations.

The SPICE model developed for the p-/n-type OECTs in this work is an equivalent circuit, consisting of two resistors (R1, R2), one capacitor (C1), one diode (D1), and one p-type or n-type MOS transistor (Supplementary Figure 6). Both static and transient characteristics (especially for the asymmetric rise/fall time) of the printed OECTs are well simulated by the equivalent circuit model. Capacitance C1 is chosen to incorporate the experimentally obtained rise times of the transistors in the simulation. Although not a direct interpretation of the overlap capacitance, both C1 and overlap capacitance (parasitic capacitance) result in similar effects, i.e., an increase in the OECTs' rise time. Hence, because of the correlation between rise time and frequency, it can be used to predict the neuron firing frequency.

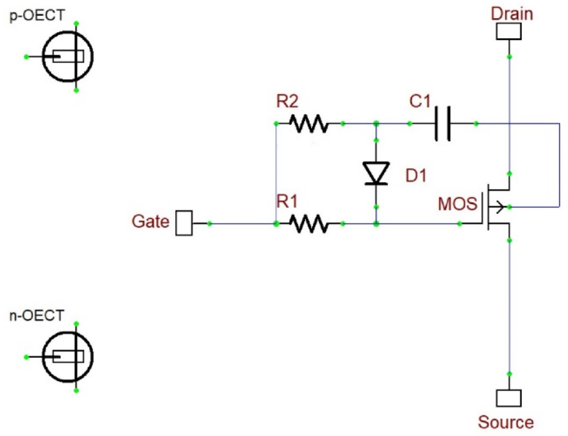

Supplementary Figure 6. Both p-type (p-OECT) and n-type (n-OECT) OECTs are built as an equivalent circuit (right) in SPICE. The equivalent circuit consists of two resistors (R1, R2), one capacitor (C1), one diode (D1), and one p-type or n-type MOS transistor (level=1).

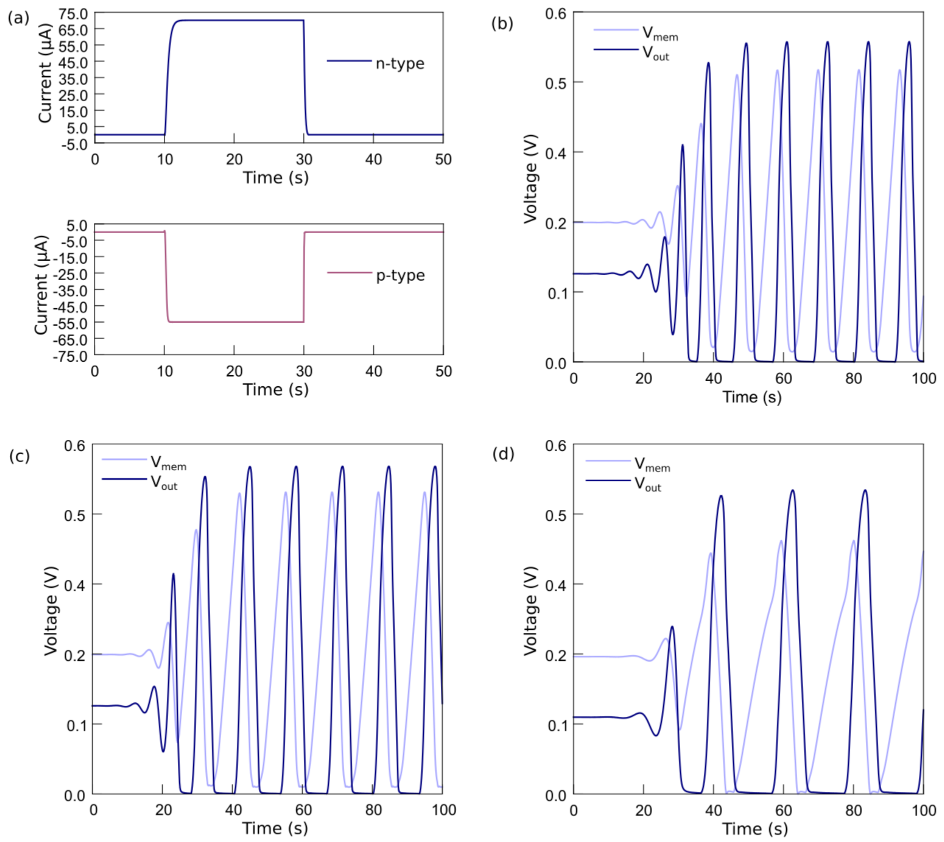

Supplementary Figure 7. (a) Simulated transient behaviors of p-type and n-type OECTs. Simulated OECN spiking firings (V_{mem}) and output of the Amplifier A (V_{out}) with three different capacitances C_{mem} and C_f, 100 nF (b), 1 μF (c), and 6.8 μF (d), respectively with 1 μA input current.

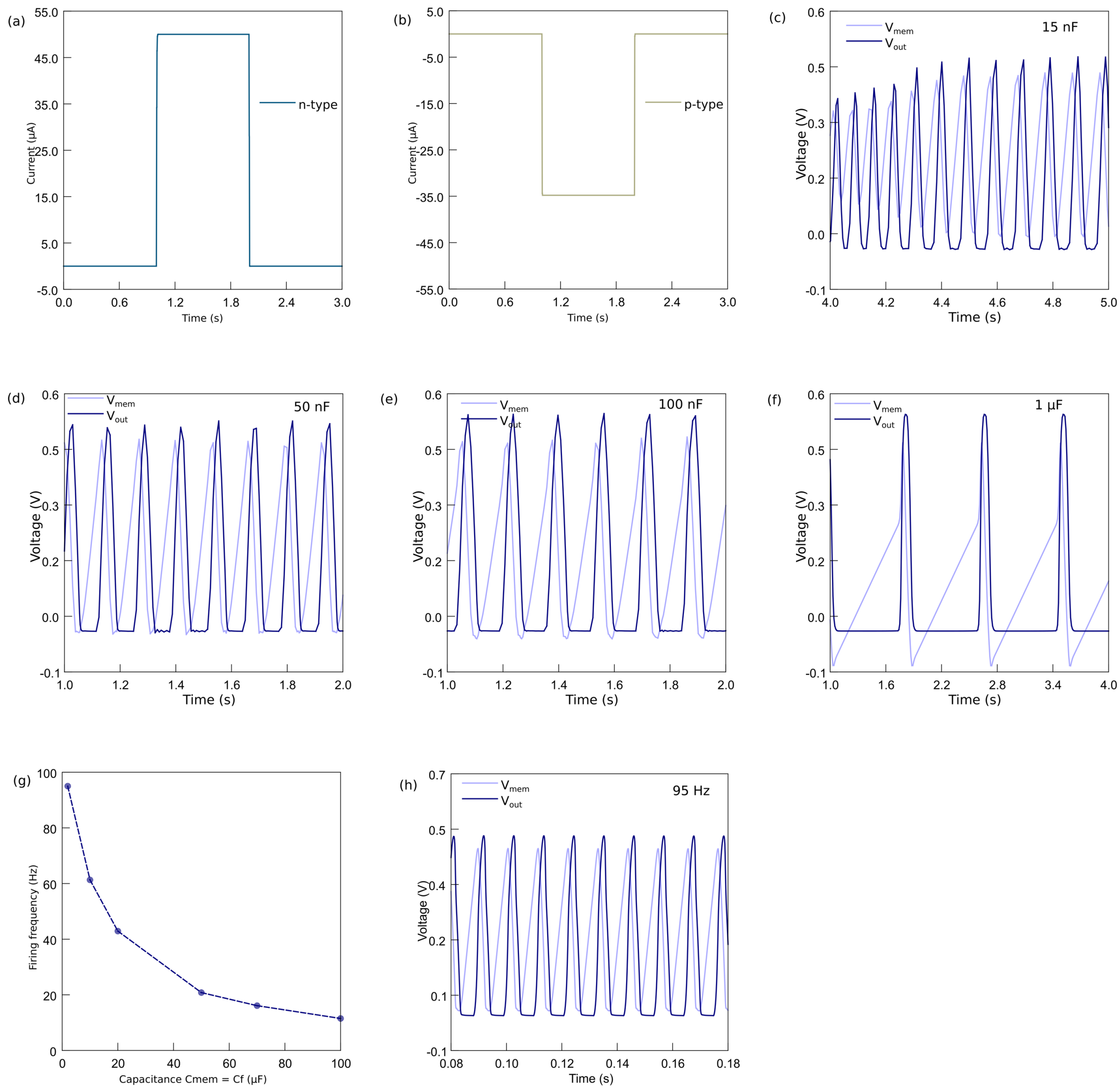

Supplementary Figure 8. (a,b) Simulated transient behaviors of p-type and n-type OECTs with fast switching speed (t_r = 0.5 ms). The simulated OEN spiking firings (V_{mem}) and the output of the Amplifier A (V_{out}) with three difference C_{mem} and C_f, 15 nF (c), 50 nF (d), 100 nF (e), and 1 μF (f), respectively at a current input of 1 μA and switching speed of 1 ms. The simulated firing frequency depending on the capacitance at 1 μA input current and 0.5 ms rise time is summarized in (g). (h) With a fast switching (t_r = 0.5 ms), the firing frequency reaches 95 Hz at an input current of 1 μA and $C_{mem} = C_f$ = 2nF.

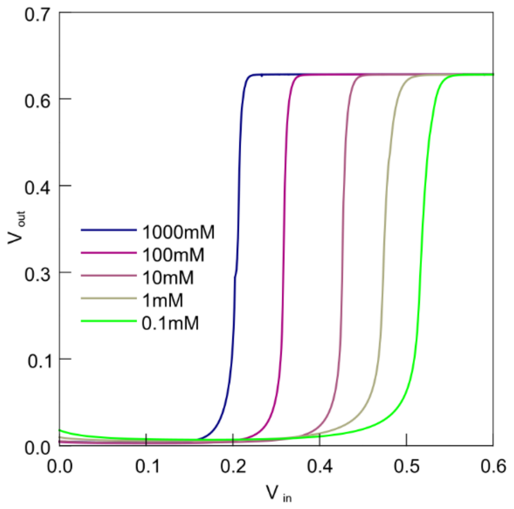

Supplementary Figure 9. Modulation of amplifier transition voltage as a function of the ion concentration in the electrolyte.

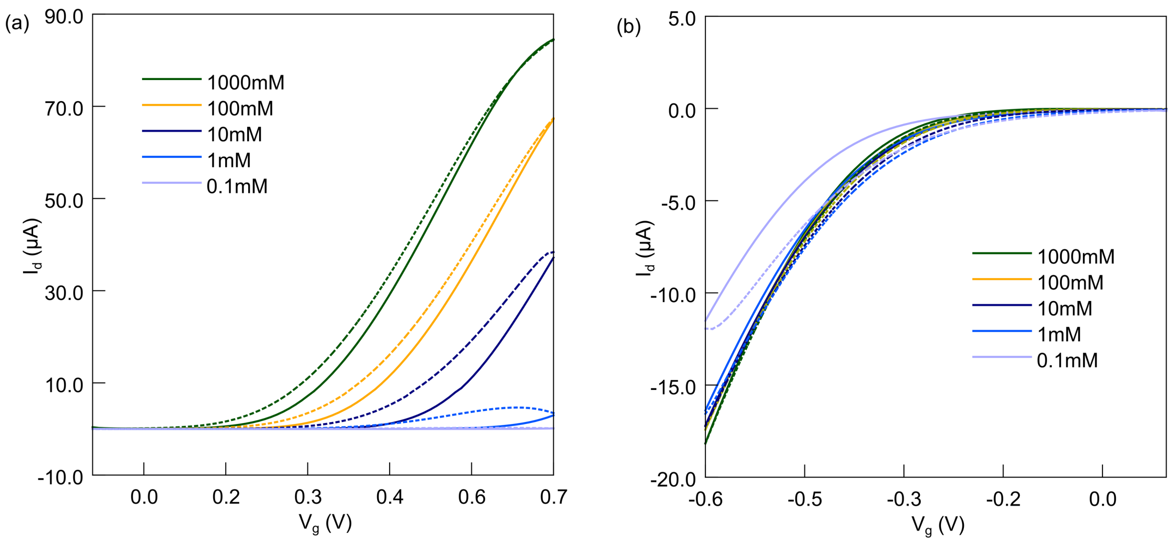

Supplementary Figure 10. Forward and reverse sweep transfer curves of (a) n-type BBL and (b) p-type P($g_4$2T-T) at different concentration of electrolyte.

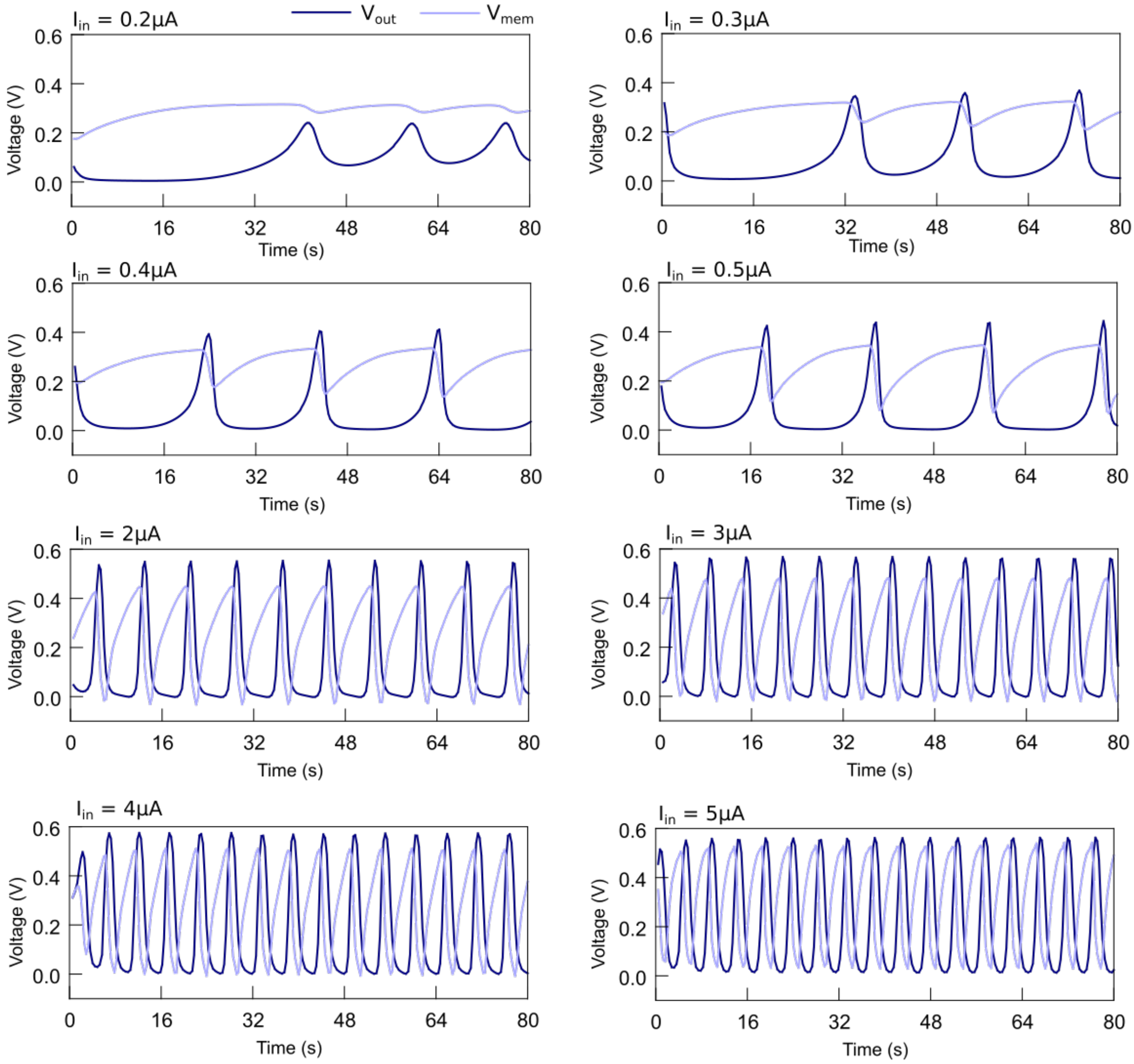

Supplementary Figure 11. Neuron characteristics at various input currents for C_{mem} = C_f = 100 nf

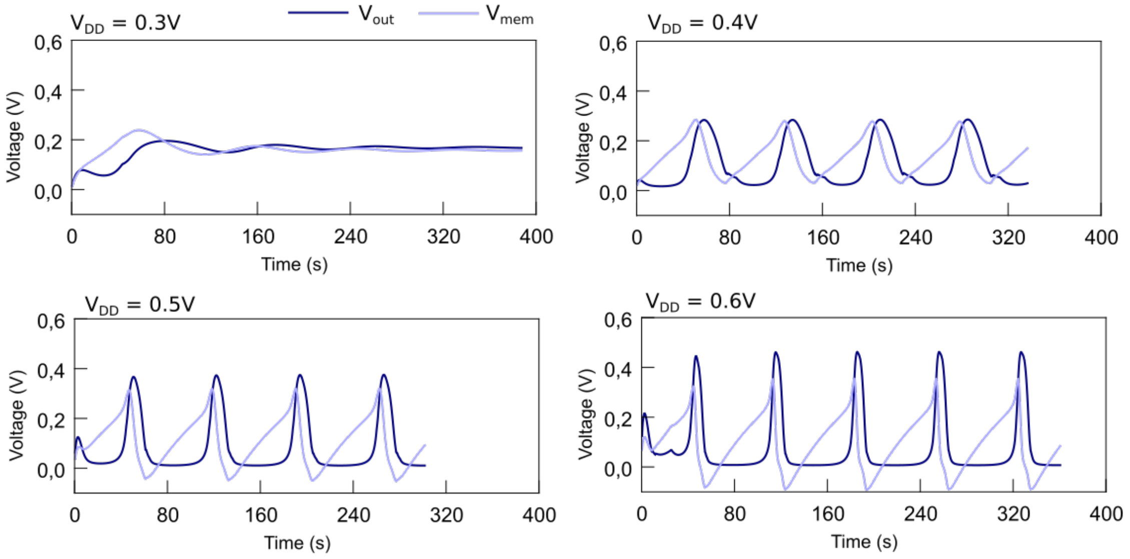

Supplementary Figure 12. Neuron characteristics at various V_{DD} values at a constant input current of 1 μA and $C_{mem} = C_f = 100$ μF

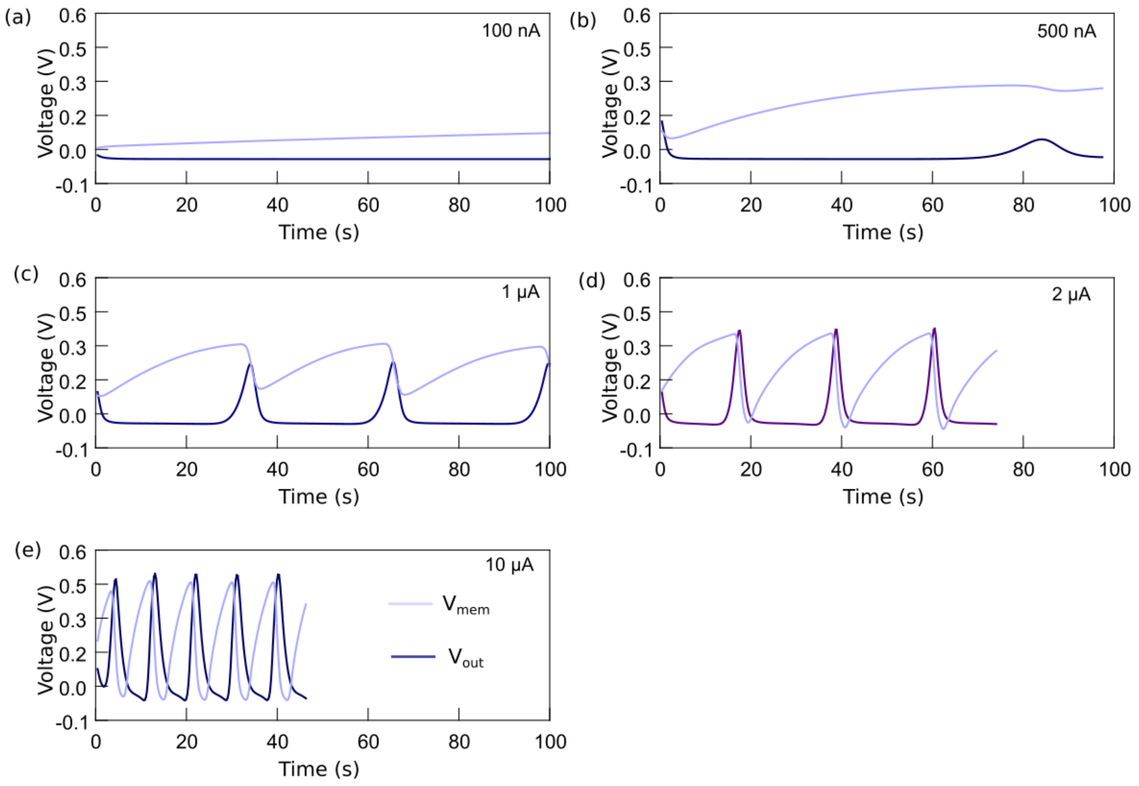

Supplementary Figure 13. Spiking frequency of the fully printed OECN for various input currents.

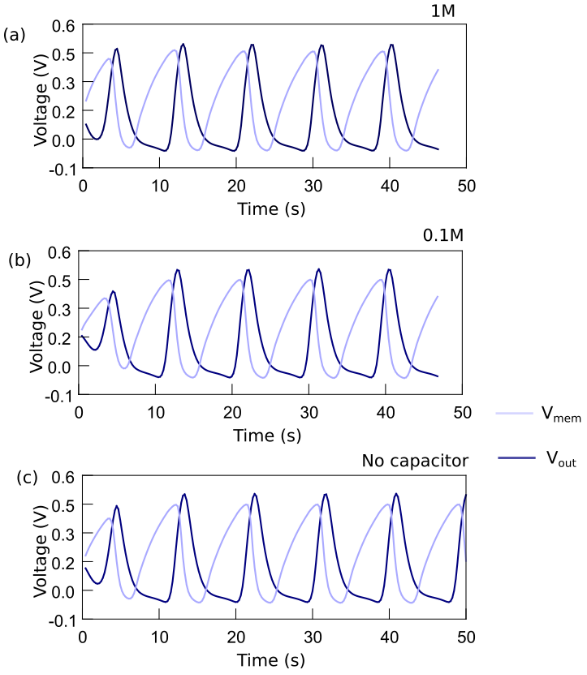

Supplementary Figure 14. Spiking frequency at 10 μA input current with various concentrations of electrolytes in the capacitors and absence of external capacitor .

Supplementary Note 3: Stability of the OECNs.

We observed no changes in the spiking frequency of OECNs on storage in the ambient for 3 months, although the voltage output reduced by 0.1 V (Supplementary Figure 15a,b). Even on continuous operation for over 1 h (Supplementary Figure 15c), we observed minimal (< 10 %) drift in the frequency of operation. We noticed a slight instability in the output current of the p-type OECT over prolonged operation (1 h), resulting in a voltage drop. This only causes a minor change in the final spiking response of OECNs as the frequency of spiking is mainly determined by the capacitances and the current/speed of the resetting n-type BBL OECT, which is instead remarkably stable[5]. This high tolerance of the circuit towards OECTs' current mismatch makes the OECNs ideal for bio-integration. Furthermore, in a real scenario, there is rarely a need for such continuous spiking of the neuron as event-based learning circuits with spiking neurons are generally triggered only when there is a sensory input.

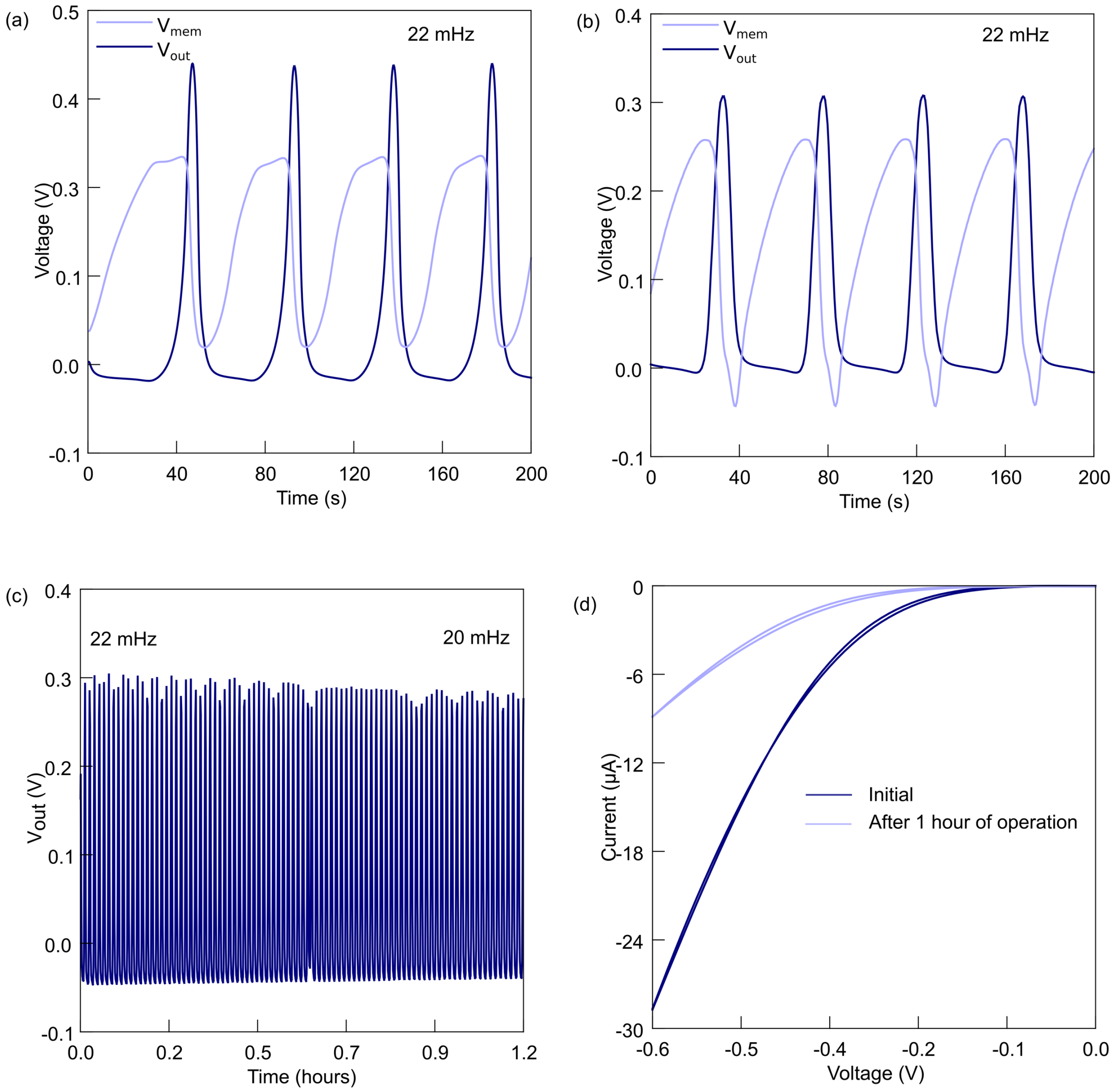

Supplementary Figure 15. (a) Initial spiking response of the OECN at 1 µA input current. (b) Response after storage in ambient for 3 months. (c) Continuous operation of the OECN for 1 h. (d) Transfer characteristics (at -0.6 V drain voltage) of the p-type OECT before and after 1 h of continuous operation.

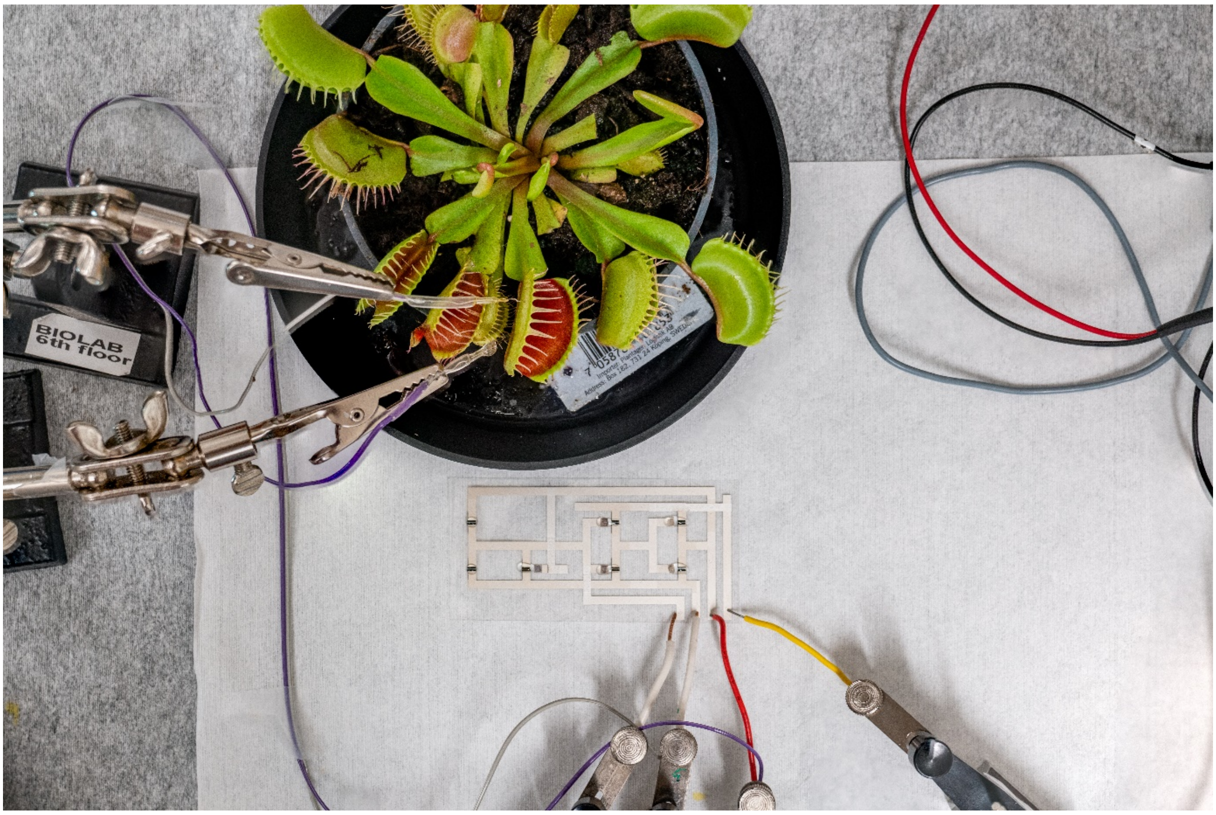

Supplementary Figure 16. Experimental setup for interfacing OECN with the Venus Flytrap (VFT). The resistance of the wires used to connect the OECN with VFT were < 1 Ohm and do not affect the OECN output.

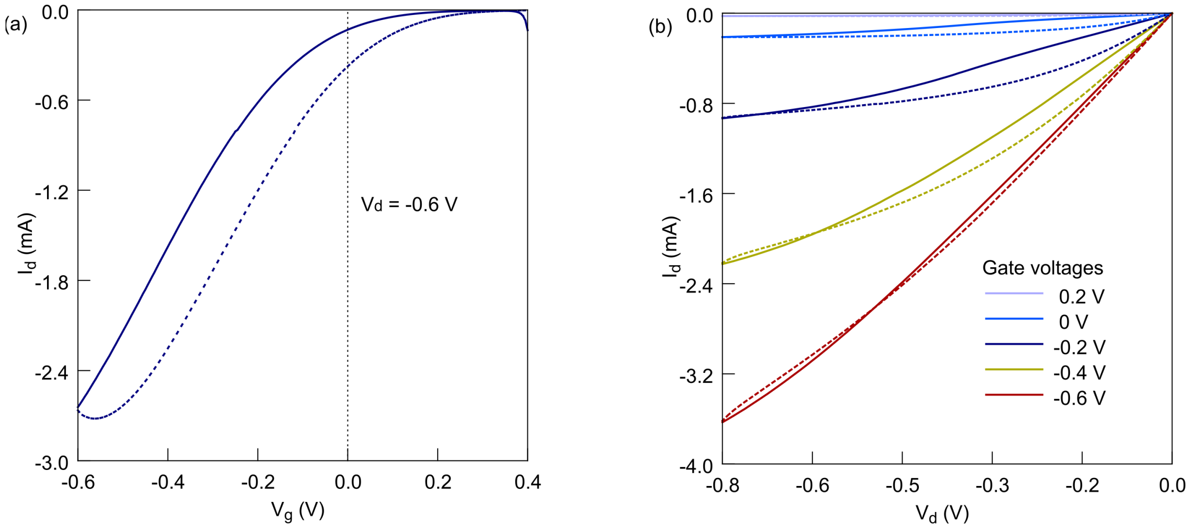

Supplementary Figure 17. (a) Transfer and (b) Output characteristics of ETE-PC synapse. Solid lines show forward sweep and dotted lines represent reverse sweep.

Supplementary Note 4: Retention of states in electropolymerized films and reversibility.

The electropolymerized layers can lead to longer-lived learning (Supplementary Figure 18). We collected data for the retention of a particular state over 15 h and found a < 1.5 % change in the current level. This is comparable to state-of-the-art OECT based synapses exhibiting long term learning[9,10]. The exact reasons for the slight increase in current observed over 15 h measurement remains elusive at the moment but could be due to background polymerization happening in the film. This change may be reduced by using radical quenchers to stop the reaction or by suitable material engineering approaches to tune the energy levels of the monomer to increase the activation energy required for polymerization.

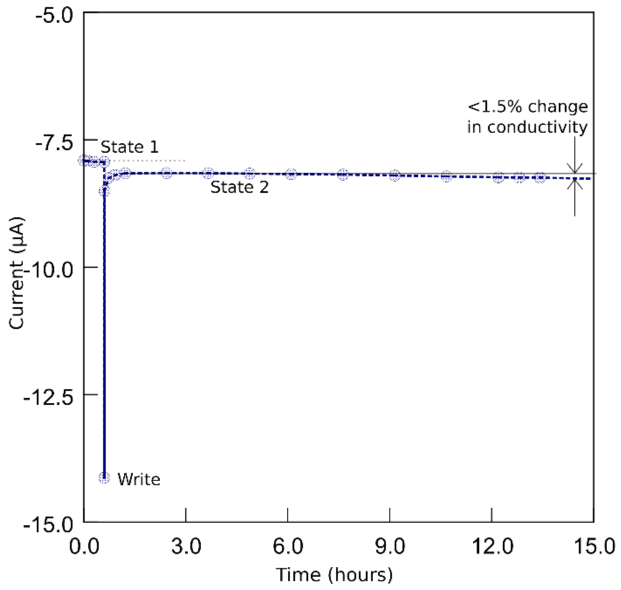

Supplementary Figure 18. Stability of a particular state over 15 h. The write pulse is a 2 s-long -0.6 V gate voltage and the current is read using a -10 mV drain voltage. The OECT was covered with 2 mM ETE-PC and 10 mM NaCl and an initial channel was formed and kept in ambient for around 1 week before starting the measurement to allow the system to stabilize.

In-situ electropolymerization is not reversible in the conventional sense where the polymer is converted back to the original monomers. However, the formed polymer can be over-oxidized to reduce its conductance, either incrementally or completely, as shown in our previous study[11]. The over-oxidized monomer is brittle and can be washed away, so the synapse can thus be fully regenerated. However, the overoxidation process has to be carried out without the monomer

solution, which can be a bottleneck in achieving facile and fast reversibility. As an alternative strategy to induce reversibility, we observed that the application of a high positive gate potential (> 2 V) can also lead to permanent reduction in conductivity of the polymer and this process seems to be reversible (Supplementary Figure 19). This process is reasonably fast and can be achieved with 400 ms pulses in a device with L = 100 µm and W =2000 µm. The origin of this permanent reduction in conductivity leading to long term depression is not yet clear and is under investigation.

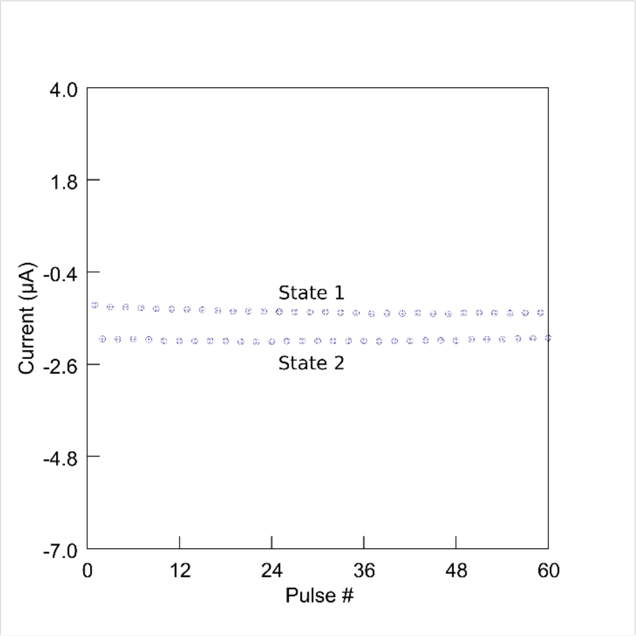

Supplementary Figure 19. 30 cycles of polymerization and reversal with alternate -0.82 V and 2 V gate write pulses of 400 ms duration. The states are read using a -0.05 V drain pulse at 0 V gate voltage, 9 s after the writing pulse.

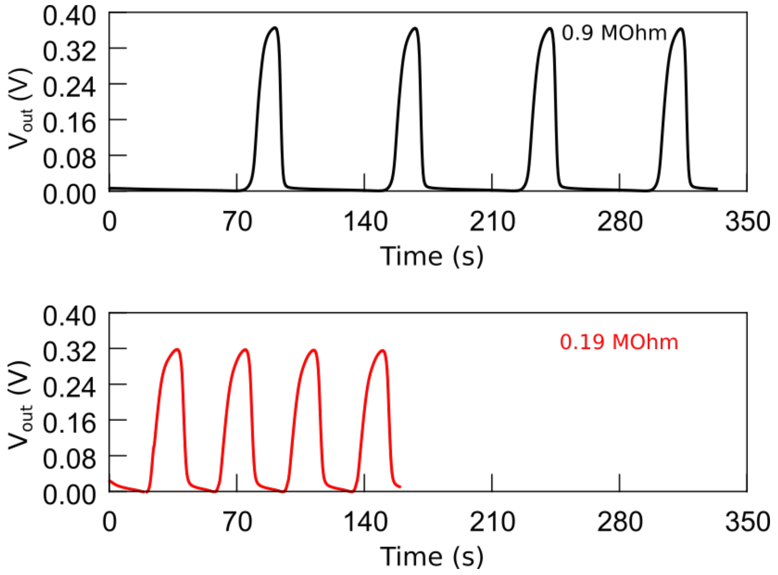

Supplementary Figure 20. Variation in spiking frequency of an OECN for two different conductivities of the OECS.

Supplementary References

1. Moshtagh-Khorasani, M., Miller, E. W. & Torre, V. The spontaneous electrical activity of neurons in leech ganglia. *Physiological Reports* **1**, (2013).
2. Baddeley, R. *et al.* Responses of neurons in primary and inferior temporal visual cortices to natural scenes. *Proceedings of the Royal Society of London. Series B: Biological Sciences* **264**, 1775–1783 (1997).
3. Macefield, V. G. & Wallin, B. G. Physiological and pathophysiological firing properties of single postganglionic sympathetic neurons in humans. *Journal of Neurophysiology* **119**, 944–956 (2018).
4. Wang, B. *et al.* Firing Frequency Maxima of Fast-Spiking Neurons in Human, Monkey, and Mouse Neocortex. *Front Cell Neurosci* **10**, 239 (2016).

5. Wu, H.-Y. *et al.* Influence of Molecular Weight on the Organic Electrochemical Transistor Performance of Ladder-Type Conjugated Polymers. *Advanced Materials* **n/a**, 2106235.
6. Kusaka, Y., Fukuda, N. & Ushijima, H. Recent advances in reverse offset printing: an emerging process for high-resolution printed electronics. *Jpn. J. Appl. Phys.* **59**, SG0802 (2020).
7. Resnick, D. J. & Choi, J. A review of nanoimprint lithography for high-volume semiconductor device manufacturing. *Advanced Optical Technologies* **6**, 229–241 (2017).
8. Zhang, S. *et al.* Synergistic Effect of Multi-Walled Carbon Nanotubes and Ladder-Type Conjugated Polymers on the Performance of N-Type Organic Electrochemical Transistors. *Advanced Functional Materials* **n/a**, 2106447.
9. van de Burgt, Y. *et al.* A non-volatile organic electrochemical device as a low-voltage artificial synapse for neuromorphic computing. *Nature Materials* **16**, 414–418 (2017).
10. Ji, X. *et al.* Mimicking associative learning using an ion-trapping non-volatile synaptic organic electrochemical transistor. *Nat Commun* **12**, 2480 (2021).
11. Gerasimov, J. Y. *et al.* An Evolvable Organic Electrochemical Transistor for Neuromorphic Applications. *Advanced Science* **6**, 1801339 (2019).
