# Supplementary material for: Organic electrochemical neurons and synapses with ion mediated spiking
Source: Nat Commun. 2022 Feb 22;13:901. doi: 10.1038/s41467-022-28483-6 (PMC8863887; doi:10.1038/s41467-022-28483-6)
Supplement: Supplementary file 2 — Description of Additional Supplementary Files [file 41467_2022_28483_MOESM2_ESM.pdf]

Description of Additional Supplementary Files

File Name: Supplementary Movie 1
Description: Modulation of the Venus flytrap with a high input current (10µA) to the fully printed artificial neuron : The firing frequency is high and the fly trap closes on stimulation with two spikes.

File Name: Supplementary Movie 2
Description: Modulation of the Venus flytrap with a low input current (2µA) to the fully printed artificial neuron : the fly trap remains open as the firing frequency of neuron is low.
